# Alternative Approaches to Vibration Measurement Due to the Blasting Operation: A Pilot Study

**DOI:** 10.3390/s19194084

**Published:** 2019-09-21

**Authors:** Stanislav Kepak, Martin Stolarik, Jan Nedoma, Radek Martinek, Jakub Kolarik, Miroslav Pinka

**Affiliations:** 1 Department of Telecommunications, Faculty of Electrical Engineering and Computer Science, VSB-Technical University of Ostrava, 17. listopadu 15, 708 33 Ostrava-Poruba, Czech Republic; 2 Department of Geotechnics and Underground Engineering, Faculty of Civil Engineering, VSB-Technical University of Ostrava, Ludvika Podeste 1875/17, 708 33 Ostrava-Poruba, Czech Republic; 3 Department of Cybernetics and Biomedical Engineering, Faculty of Electrical Engineering and Computer Science, VSB-Technical University of Ostrava, 17. listopadu 15, 708 33 Ostrava-Poruba, Czech Republic

**Keywords:** blasting operation, seismic measurements, interferometric sensor, fiber-optic sensor, acoustic sensor

## Abstract

As the infrastructure grows, space on the surface in the urban area is diminishing, and the view of the builders is increasingly moving underground. Implementation of underground structures, however, presents a number of problems during construction. One of the primary side effects of tunnel excavation is vibration. These vibrations need to be monitored for potential damage to structures on the surface, and this monitoring is an integral part of any such structure. This paper brings an original pilot comparative study of standard seismic instrumentation with experimentally developed fiber-optic interferometric and acoustic systems for the purpose of monitoring vibration caused by the blasting operation. The results presented show that systems operating on physical principles (other than those previously used) have the potential to be an alternative that will replace the existing costly seismic equipment. The paper presents waveform images and frequency spectra from experimental measurements of the dynamic response of the rock environment, due to blasting operation performed shallowly during the tunnel excavation of a sewer collector. In the time and frequency domain, there is, by comparison, significant agreement both in the character of the waveform images (recording length, blasting operation timing) and in the spectra (bandwidth, dominant maxima).

## 1. Introduction

Monitoring vibration generated during the blasting operation is a standard discipline of seismic and geotechnical engineering. This monitoring is carried out during the implementation of each underground construction driven by cyclic excavation, i.e., the alternation of drilling, blasting, excavation and reinforcement operations, especially when the construction is created shallowly, under the surface of a built-up area. Civilian and civic buildings that may be endangered as a result of excavation operations or vibration generated are monitored. The specific requirements for these measurements are always defined by the project of geotechnical monitoring of the structure, and the assessment of dynamic effects is then carried out on the basis of national standards [1,2,3,4,5]. 

For seismic effects, vibration amplitude (measured as the size of velocity or acceleration) and frequency content, or dominant signal frequency, are commonly monitored. The wide spectrum of frequencies is then dependent on the properties of the material to be disintegrated, the properties of the explosive used and the technology of the blasting operation to be performed. The frequency spectrum of the blasting operation seismic recording is also significantly influenced by the environment in which the waves propagate. With increasing distance, the components of higher frequencies are damped faster in the rock mass. The dependence of the frequency range of the seismic signal on the distance from the blasting operation implies that it is necessary for the seismic channel to have the widest possible frequency range at short distances from the blasting site. The frequency spectrum of seismic signals induced by the near blasting operation in rocks may contain frequencies from 1 to 300 Hz [6,7]. Also, the monitoring of acoustic noise from blasting operations, both in the excavation of underground works and in the extraction of mineral resources, is necessary according to hygiene regulations and legislation (for example, based on the regulation entitled Coll. on health protection against adverse effects of noise and vibration [8]).

For decades, piezoelectric seismometers have been used as a standard for vibration monitoring [9]. However, in the rapidly and progressively developing the 21st century, there are other physical principles that can be used to measure vibration and that have a high added value, such as low prices, simple interior construction, mechanical resistance, possibility of being used in extreme conditions (danger of explosion, extreme temperatures, humidity, etc.). 

The paper is a pilot study that is to show alternative possibilities of measuring vibration caused by blasting operations using other physical principles than have been commonly used so far. In the paper, the original results obtained from the pilot measurements using an experimentally developed fiber-optic interferometric sensor and an experimentally developed acoustic sensor when detonating a small amount of explosive during the excavation of a sewer gallery are compared with the results obtained from standard instrumentation for seismic monitoring, both in the amplitude and frequency range. It was an experiment in the so-called near zone, i.e., within the first tens of meters from the dynamic load [10], and the dynamic response of the rock mass was monitored.

The fiber-optic interferometers dispose of many valuable properties, such as their small size, resistance to electromagnetic interference (EMI), electrical passivity and low attenuation, providing to separate the measuring point from the point of evaluation. The fiber-optic interferometers, such as the Mach-Zehnder, allow high-precision measurements of optical path’s differences, alterations caused by the change of a refractive index in the interferometer or physical displacement [11].

The summary of the current research within fiber-optic technology application and in case of the general interferometric measuring approach is stated below. The current trend in case of recording vibration-acoustic signals using the fiber-optic technology is aimed partially to the area of natural seismic activity and also to the transport (vibration-acoustic responses from road/railway vehicles).

State of the art in the area of seismic measurements mentions the possibility to apply the interferometric approach of measuring for about two decades ago [12]. These are, however, initial indications not based on the fiber-optic technology. Application of the distributed fiber-optic technology [13] called DAS or DVS (Distributed Acoustic/Vibration Sensing) which use interferences on Rayleigh backscattering has already proven to be an alternative approach. These technologies enable to scan the acoustic signal and vibration over the total length of the optical fiber, which has no alternative in the conventional measuring technology. The disadvantage of this method is the high price (hundreds of thousands of dollars) of the evaluation unit and also difficult measuring of wave direction at the measuring point. For these reasons, it has not taken on yet in practice, and it is used rather on an experimental basis.

In the case of fiber-optic approach within the interferometric measuring, Sagnac interferometers [14] were described, which, however, apply in practice mainly as gyroscopes to date. The ability to measure rotation during seismic activity proved interesting also because it enables measuring even with the use of coherent radiation sources contrary to other types of interferometers.

Fabry-Perot, Mach-Zehnder or Michelson fiber-optic interferometers are operating on the principle of acceleration detection [15] distinguish themselves by capabilities to substitute seismic stations. Its characteristic output is then the intensity modulation measurable with standard optical power detector, which in principle may lead to functionally comparable solutions. As apparent from the latest research [16], sensors with these types of interferometers can be constructed as tri-axial; however, such sensor is not built as all-fiber, since interferometers monitor the position of pendulums only. By contrast, our proposed solution should be all-fiber, which would also simplify the sensor structure (more compact), while preserving sufficient sensitivity.

Several studies deal with a non-fiber interferometric measuring approach. The impressive results reported the monitoring of bridge statics and dynamic vibrations in [15], where two different camera types monitored the response of a bridge to a passing train. Three different image processing methods were used for the analysis of the acquired images: Pattern matching, edge detection, and digital image correlation. Then, the comparison of the results and the reference measurements using laser interferometer, allowing single-point assessment, was made. The use of in-fiber Fabry-Perot interferometer with fiber-Bragg grating mirrors (FBG-FPI) was proposed in [16]. This type of sensor was recommended by the authors to monitor a wide range of vibration frequencies, such as monitoring of seismic responses. In [17], the authors compared and evaluated the suitability of two methods of geodetic measurements: Interferometric, using IBIS microwave coherent radar, and tachymetry, using Leica TC2002. The experimental results of the monitoring of dynamic changes produced by passing trains could be used to determine the alterations in the geometric condition of buildings and engineering structures. The low-frequency ground motion on seismic, geodetic, and intermediate time scales can be detected by laser interferometer using a method proposed in [18]. The authors of [19] deal with the geophysical response of fiber-optic acoustic sensors in laboratory-scale experiments, where the acoustic data recorded simultaneously using a fiber-optic interferometer (Michelson type) and conventional three-axial accelerometers were analyzed.

Among other things, the team of authors has already dealt with the use of the Mach-Zehnder interferometer to measure the dynamic effects caused by tram, railway and road transport, as well as the monitoring of the dynamic effects of the construction technology, and the applicability of fiber optic sensors to the monitoring of these effects has been confirmed at the experimental level and with some limitations. [20,21]. This research is still ongoing [22].

In the case of fiber-optic technology, in addition to the interferometric measurement approach, it is necessary to mention approaches in the form of fiber Bragg grating (FBG) [23,24,25,26,27] or distributed systems like distributed strain and temperature sensing (DSTS) [28,29,30,31]. However, both technologies work on a different principle (against our presented solutions) and bring some advantages and disadvantages in the field of civil engineering.

A unique acoustic device that is being developed was also used for vibration measurement. This sensor works as a converter of physical quantities and, according to the research performed, it has no analogy in its area, and there is no commonly used name for it. This device is most similar to closed-hose presence detectors that detect objects by direct application of their weight to the tube and its subsequent deformation and pressure change [32,33,34]. In contrast to this method, the device presented operates on the basis of measuring micro-vibrations propagated by the surrounding material in the form of longitudinal waves. Another similar and inverse principle is the measurement of the pressure, density, or flow rate of a medium through the tube-based on vibration measurement [35,36,37]. In these cases, the vibration is caused by the deformation of the tube and the effect of the medium, as opposed to our case where the vibration affects the tube and the pressure inside it. 

## 2. Methods

### 2.1. Seismic Equipment BRS32

**Figure 1:**
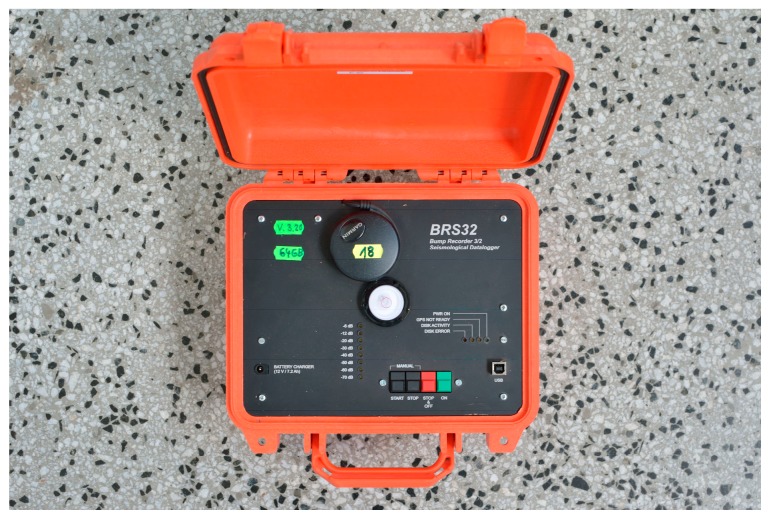
Universal seismic apparatus BRS32.

This BRS32 is universal seismic apparatus that can be used for a wide range of seismic measurement applications using battery power connection in field conditions, as well as for long-term seismic monitoring using electrical network connection and remote data transfer (LAN), see Figure 1. The device is equipped with a three-component seismic geophone, and the recording is made on a 64 GB internal flash memory. Data downloading and other parameters are configurable via a USB interface. The frequency range and the dynamic range depending on the internal geophone installed and lie between 0.5 Hz and 80 Hz at the dynamics of up to 120 dB. The recorder itself has a dynamic input range greater than 144 dB. After switching on, the equipment is automatically connected to the Global Positioning System (GPS) signal, which ensures time synchronization of the data with high accuracy (depending on the sampling rate selection) and saves the measurement coordinates. It worked for more than 48 h on a single charge of the internal battery. Due to the overall size and simple manipulation, the apparatus is suitable for all induced and natural seismicity measurement applications. Processor equipment of the device also enables synchronous seismic measurement with GPS time identification in underground spaces when synchronizing the time channel before entering the underground and re-synchronizing when leaving the underground and capturing the GPS in the free space. The equipment is mass-produced in the Czech Republic. Internal sensory equipment can be selected, depending on the character of the measurement, either with Dutch SM6 internal geophones (4.5 to 100 Hz) or with 1 Hz LE3D sensors made by the German company Lennartz (1 to 80 Hz), or another external sensor can be connected [38]. In this particular equipment used, a geophone SM6-3D (EGL Equipment services, Xi’an, China) is installed. 

### 2.2. Experimentally Developed Fiber-Optic Interferometric Sensor

An interferometer is a device operating with a radiation source whose waves are divided into two or more parts, which are then put together to form interference. This phenomenon can also be observed in waveguides. The phase delay of light, due to passing through the optical fiber section, is given by relation (1), where $n_{0}$ is the refractive index of the fiber core, l represents the fiber length and λ the wavelength of the radiation used.

(1) $$\Phi =\;kn_{0}l\;=\;\frac{2\pi n_{0}l}{\lambda },$$

Interferometers can be used to measure phase changes between the individual waves, wherein these changes are translated into intensity changes, so they can be detected by conventional power photodetectors. The basis of the sensor function is the sensitivity of the phase, as one of the characteristics of light waves, to the external conditions with which the optical fiber comes into contact. This is, according to the theory of elasticity, sensitive to the mechanical stress acting in the fiber axis, i.e., the expansion or compression of the fiber. This results in a change in the refractive index of the core and the cladding of the fiber, its length and, to a lesser extent, the diameter of the fiber core [39].

**Figure 2:**
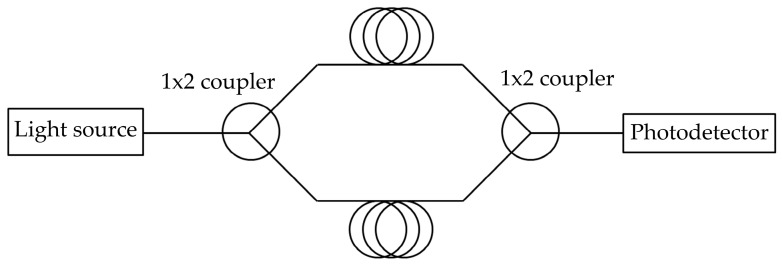
A basic connection of the Mach-Zehnder interferometer.

In the basic connection of the Mach-Zehnder interferometer (MZI), in its fiber-optic version, there are two couplers in a configuration with either one or two input ports and two output ports. An optical radiation source is connected to the input port of the first coupler, the two output ports and the short fiber segments form the measurement or reference arms of the interferometer. These are connected to the two inputs of the second coupler, and its output or outputs are then fed to the photodetector(s), see Figure 2. The sensor output can then be described by Equation (2):

(2) l(t)= C+Acos(ΔΦ(t)), 

where *C* is the mean value of the optical intensity, *A* is the amplitude of the variation of the optical intensity, and ΔΦ(t) is the phase difference between the individual arms of the interferometer. Accurate determination of the value of ΔΦ(t) using a demodulation technique is essential for the measurement. The passive homodyne demodulation technique selected uses a coupler in a configuration with three output ports that are phase-shifted relative to each other. The calculation is then performed digitally, using the harmonic arctangent function [40].

The mechanical design of the proposed sensor is based on an IP65 plastic box having dimensions of 45 × 35 × 17 cm. For maximum vibration transmission, very hard material with Young’s modulus of tens of GPa [41] is needed, and a hole has been cut in the bottom of the box that allows the sensor is measuring fiber to be attached to a 50 × 50 cm glass base plate having a thickness of 1 cm. The connection of the plastic box and the glass plate is provided by a silane-modified polymer-based adhesive in order to maintain water tightness. A measuring optical fiber having a total length of 3 m is attached to the glass by means of an adhesive. For ideal mechanical stress transfer, the adhesive should have the Young’s modulus, again as high as possible [42]. A two-component epoxy resin was used.

**Figure 3:**
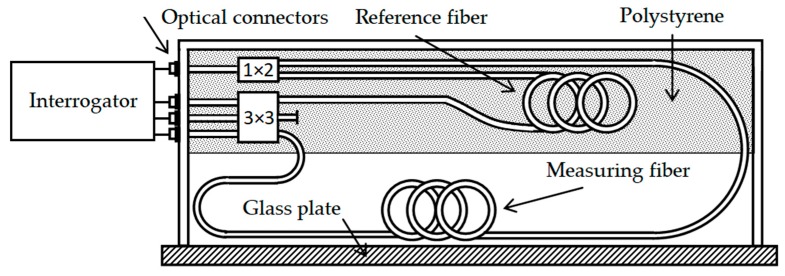
Connection of the interrogator unit with the fiber-optic interferometric sensor.

The reference fiber, also 3 m long, is placed between two 4 cm high pieces of polystyrene, which, with its other dimensions, follow the internal dimension of the box, thus, providing acoustic and vibration insulation. The couplers forming the interferometer are also located in this insulation. Their inputs, or outputs, are then led out using coupling elements situated on the front panel of the plastic box, see Figure 3. The interrogation unit consists of 1550 nm Distributed Feedback (DFB) laser operated at 1 mW of output optical power per sensor, and the inputs are connected to regular InGaAs (indium gallium arsenide) p–i–n photodetectors. Fiber patch cords used between the sensor and interrogation unit was 10 m long with single-mode G.657.A1 fiber.

**Figure 4:**
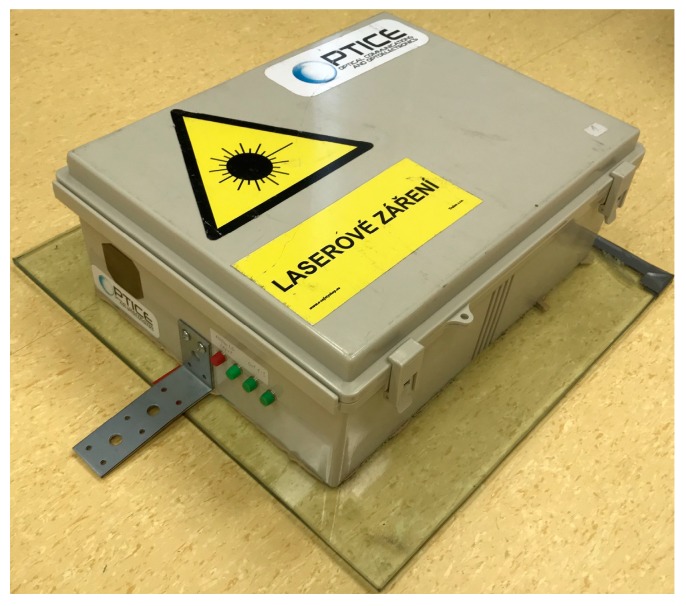
The physical version of the interferometric sensor (photo).

The output voltage signal is then amplified and led to the data acquisition module National Instruments 9202 in cDAQ-9184 (National Instruments, Austin, Texas, United States of America) chassis with the USB connection to the computer. The sampling rate was set to 10 kHz. The computer is performing the data logging, signal processing, and demodulation described above. The interrogation unit can support several fiber optical sensors in its current form. The physical sensor sample is shown in Figure 4.

The frequency characteristic of the interferometric sensor is well achieved in the range from 1 to 300 Hz (in accordance with ISO 4866: 2010). These sensitivity levels in the range from 1 to 300 Hz were almost achieved by the sensor design described above. Longer fiber segments would limit the lower cut-off frequency, due to, e.g., the temperature drift [43,44] (the drift is translated into the slow changes of ΔΦ(t)), while shorter fibers would mean lower sensor sensitivity in general.

### 2.3. Experimentally Developed Acoustic Sensor

**Figure 5:**
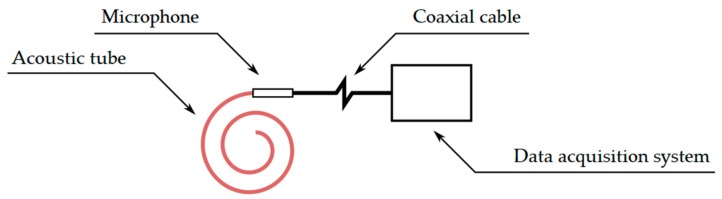
Diagram of the acoustic sensor.

Generally, this device works on the principle of vibration conversion to a change in the acoustic pressure, which is measured using a microphone (Figure 5). By its very nature, this sensor system is susceptible to changes in acoustic pressure and, commonly, other acoustic disturbances. However, this problem is solved by the construction of the sensor part.

**Figure 6:**
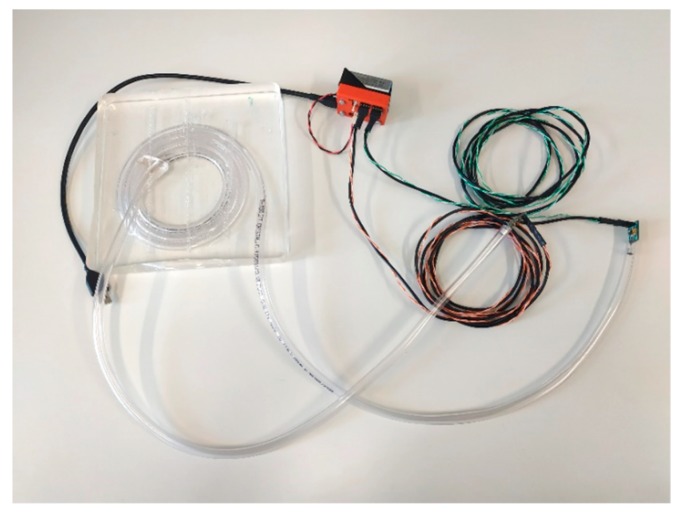
The low-cost acoustic measurement kit.

We designed our own low-cost built-in device system (Figure 6) from the following components:

- Electret microphone model PUM-5250L-R;
- Low-noise operational amplifier OPA172;
- Microcontroller unit (MCU) STM32F373;
- Sampling rate 5 kHz;
- Polydimethylsiloxane (type Sylgard 184, weight 750 g);
- Acoustic tube length of 1.5 m (PVC material).

The sensor itself consists of polyvinyl chloride (PVC) tube, which is closed on one side by a microphone and terminated with an ending on its other side. Both ends of the tube are airtight and prevent leakage of the medium. A system sealed in this manner has, at a constant temperature, a direct relationship between the volume and the pressure of the gas enclosed within the tube. One of the advantages of this system is its resistance to external acoustic interference. The sensor, or the measuring part, is encapsulated in the polymer material of polydimethylsiloxane [42]; this material increases the resistance of the sensor, is inert to EMI, high temperatures (up to 200 °C) and allows easy implementation within the measurement.

**Figure 7:**
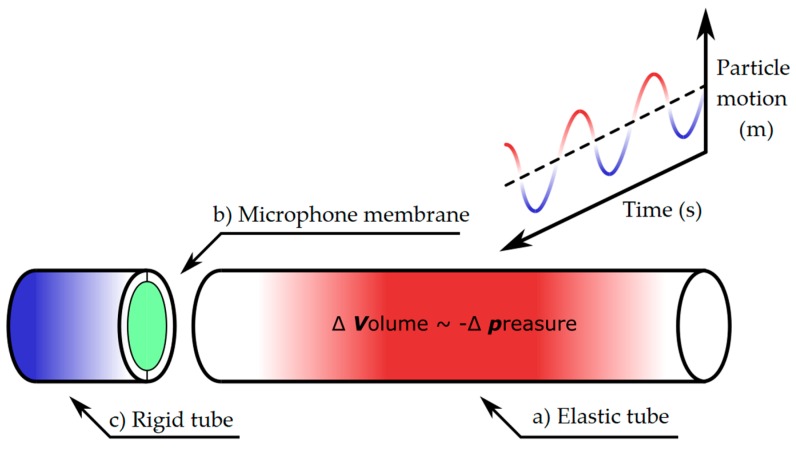
The basic principle of acoustic sensor operation.

The principle of sensor operation is shown in Figure 7. The spatial movement of the particles of the surrounding material is transferred to the jacket of the elastic tube (Figure 7a), which causes its deformation and also changes its volume. Assuming a constant temperature of the gas enclosed within the tube, there is a change in gas pressure that is inversely proportional to the change in the tube volume. This pressure change is recorded using a microphone membrane (Figure 7b). Assuming that the rigid part of the tube (Figure 7c) is an ideally rigid body, the pressure change occurs only in the flexible tube (Figure 7a). Within the damped oscillations of this system, the elastic conduit is reversely deformed (Figure 7a), and the pressure inside the conduit is stabilized.

**Figure 8:**
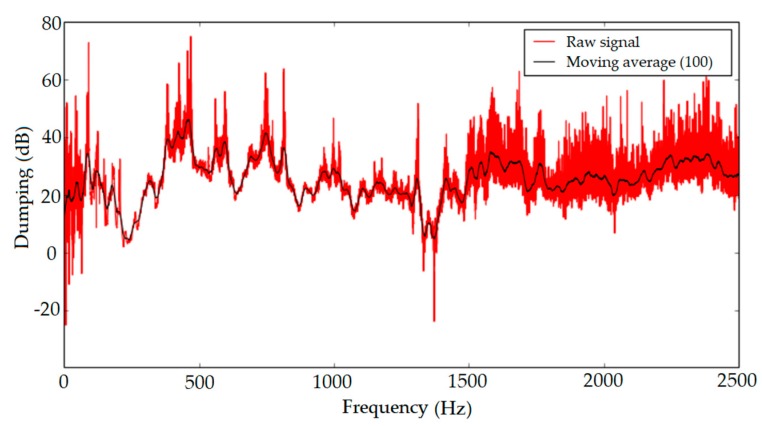
Resistance to external interferences.

To verify damping properties of the tube sensor, the measurement consisting of two identical microphones and the acoustic tube were performed. Microphones were placed inside and outside of the sealed acoustic tube, to record ambient noise and sound pervading into the acoustic tube. The results of measured dumping were around 20 to 40 dB for frequencies up to 2.5 kHz (Figure 8). 

## 3. Experimental Setup

**Figure 9:**
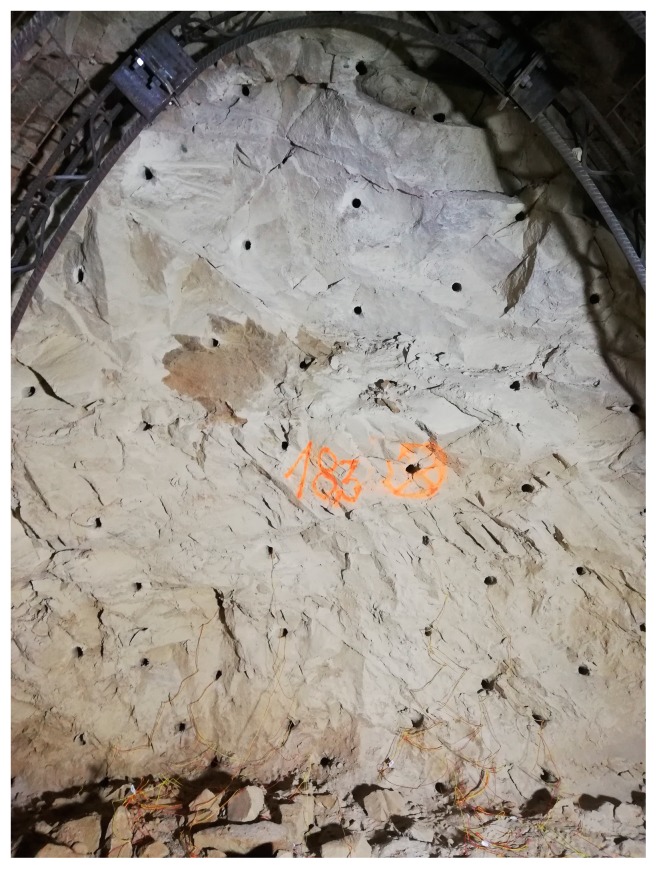
A drilled and partially charged heading.

The experimental measurements were carried out during the tunnel excavation of a sewer collector in the municipality of Radvanice, GPS coordinates: 49.8272044N, 18.3099894E. The excavation was carried out cyclically using small blasting operations with a proposed total load of 12 kg of Austrogel explosive, with a maximum effective load of 0.3 kg. The timing was performed with a total of fifty detonators DeM (Mining electric millisecond) SICCA (manufacturer’s designation for a mine safe detonator) a DeD (Mining electric half-second) SICCA and the total burden was always 1 m (Figure 9). The excavation was carried out in undamaged sandstones with sand and clay in the overburden. The groundwater level was not reached during the excavation operation. Seismic experimental measurements were conditioned by both the climatic conditions and the technological process on site. From the beginning of the excavation operation, the excavation process was accompanied by problems with overcapacity. These problems eventually resulted in the interruption of all operation in early May 2019, and a new technological procedure had to be developed. This also caused the experimental measurements to be interrupted indefinitely. For this reason, this pilot publication contains results from three measurements of the effects of blasting operations.

**Figure 10:**
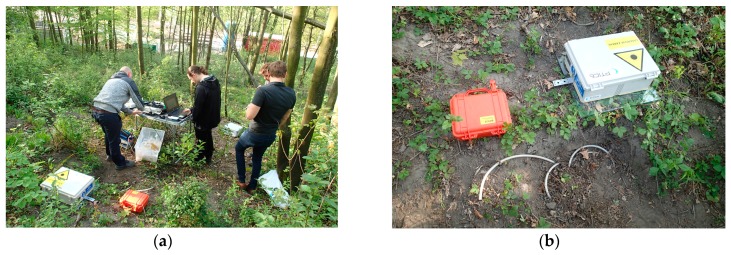
(**a**) The in-situ laboratory on 26 April 2019; (**b**) measuring instrumentation arrangement—the buried acoustic sensor in the foreground, the interferometric sensor on the right, the BRS32 apparatus on the left.

**Figure 11:**
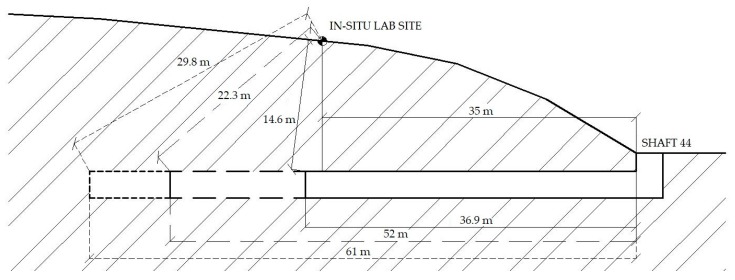
A measurement situation.

The in-situ laboratory was set up in the axis of the underground work, on a gentle slope at a distance of 35 m from the start shaft—SHIFT44 (Figure 10a). In total, three elevations were made in three blasting operations, specifically on March 11, 2019, April 8, 2019, and April 26, 2019. In those days, the heading was in the stationing of 36.9 m, or 52 m, or 61 m. The shortest possible distance of the blasting operation from the measuring station ranged from 14 m to 30 m (Figure 11). Figure 10b shows a photograph of an interferometric sensor measuring arrangement, an acoustic sensor placed in the ground to a depth of 10 cm (this depth was selected based on previous experimental tests) and the BRS32 apparatus. At each measurement, all three measuring devices were always in close proximity to each other and in the same configuration. 

### Results

This section summarizes the results of all three experimental measurements. For a better visual comparison, the waveform images and frequency spectra from the individual measuring devices and the respective days are arranged one below the other for each registered blasting operation. Due to the fact that the seismic apparatus BRS32 records in three perpendicular directions (vertical, horizontal radial and horizontal transversal), for the purpose of this pilot study, only the vertical component was compared with experimentally developed devices. 

**Figure 12:**
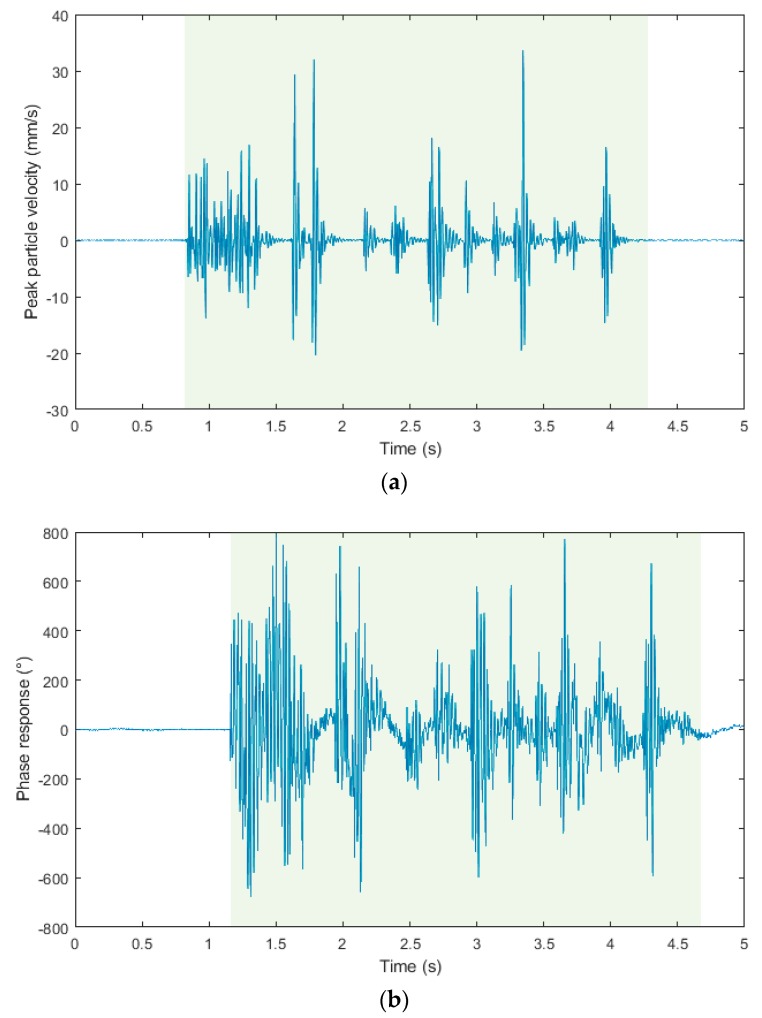

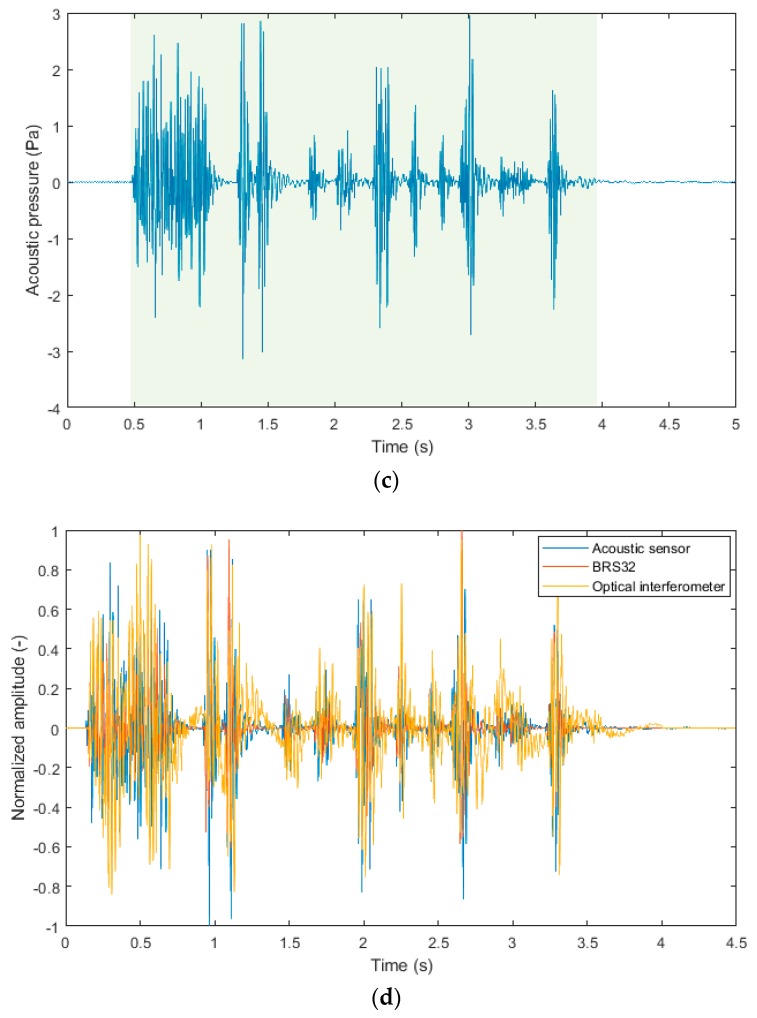
The waveform images from the blasting operation recording of 11 March 2019: (**a**) Seismic apparatus BRS32; (**b**) interferometric sensor; (**c**) acoustic sensor; (**d**) graphical comparison of the three records described above in one graph.

Figure 12a–c show time domain recordings from the blasting operation of 11 March 2019, which was located 14.6 m from the measuring station. The blasting operation recorded was three and a half seconds long, please see the light green background. This data is identical in all three recordings from the three measuring devices. The output on the Y-axis in the case of a seismic station (BRS32) is Peak Particle Velocity (mm/s), in the case of interferometric sensor Phase Response (°) and in the case of acoustic sensor Acoustic Pressure (Pa). Please note that the three signals are not time-synchronized because of the different and independent systems used. For better clarity and comparison, Figure 12d shows all signals in one graph. These signals were time-synchronized, and its amplitude was normalized so that the single dependent axis could have been used. Twenty pieces of DeM detonators with explosives in boreholes in the total amount of 6 kg and thirty pieces of DeD detonators with explosives in boreholes in the total amount of also 6 kg were used for timing. In the first second, the shot of the cut is clearly visible, followed by two distinct peaks of the first-floor ripping shot holes, and, in the following time stages, by the rest of the ripping, and, in the end, by the rim holes. All the time stages are clearly recognizable, both with the BRS32 station and the acoustic sensor. The time recording from the interferometric sensor is not quite clear, and it is blurred. This is a phenomenon that can occur in the time domain in the so-called near zone (the first meters from the dynamic load), when the sensor also detects the elastic deformation of the rock mass [45,46]. This can be removed both by design modification of the sensor and by alignment, or by firm attachment of the sensor to the base [47].

**Figure 13:**
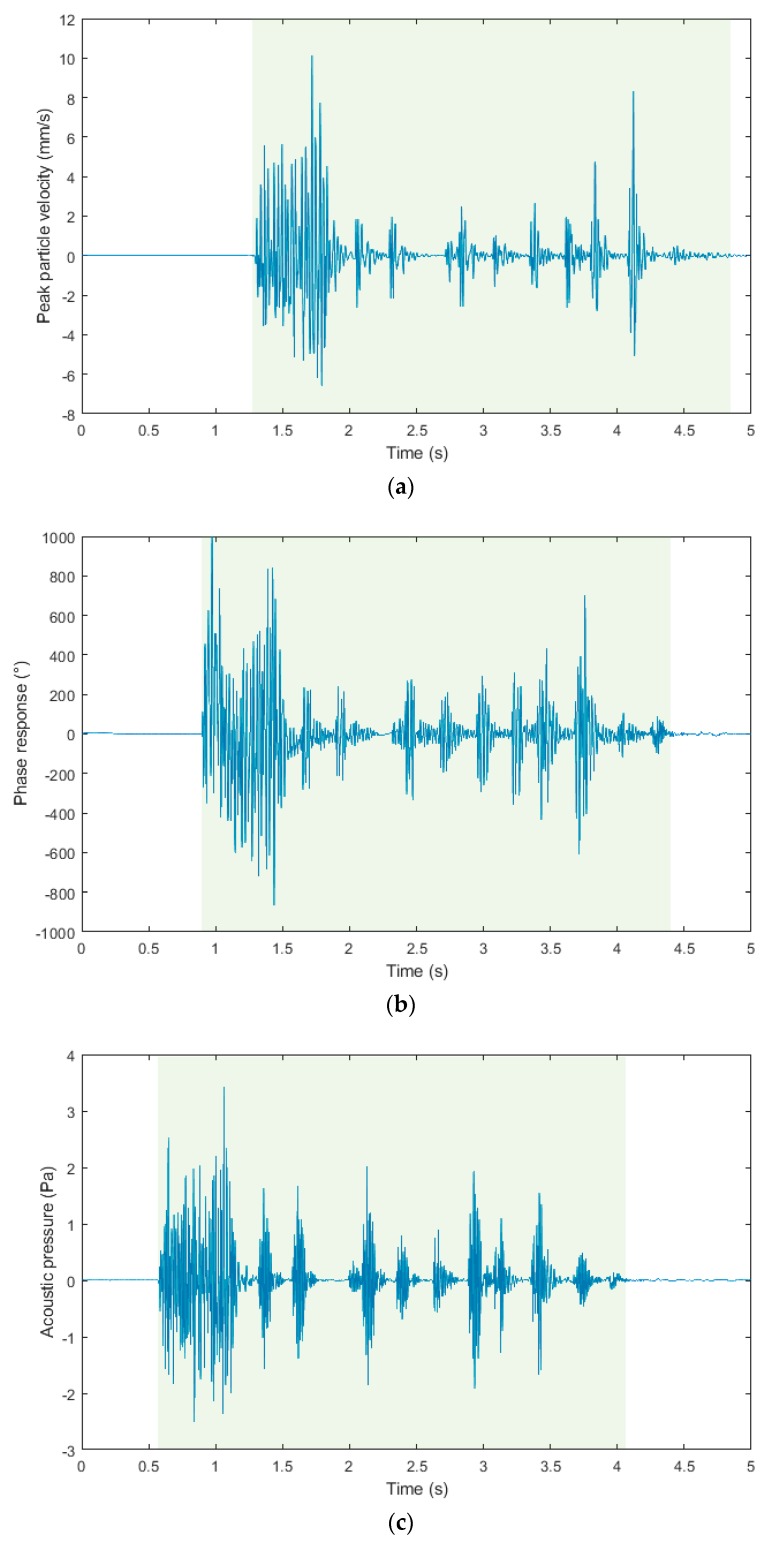

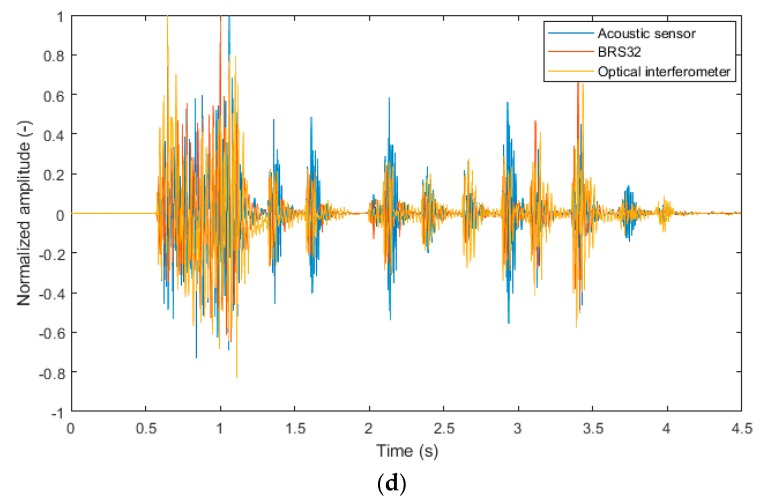
The waveform images from the blasting operation recording of 8 April 2019: (**a**) Seismic apparatus BRS32; (**b**) interferometric sensor; (**c**) acoustic sensor; (**d**) graphical comparison of the three records described above in one graph.

Figure 13a–c shows time domain recordings from the blasting operation of 8 April 2019, which was located 22.3 m from the measuring station. The blasting operation recorded was three and a half seconds long, please see the light green background, and the recording differs from the previous one because another type of timing was used. Figure 13d, again for better clarity, illustrates a graphical comparison of the three records described above in one graph. Forty pieces of DeM detonators with explosives in the total amount of 9 kg and ten pieces of DeD detonators with explosives in the total amount of 3 kg were used for timing. Again, both the shot of the cuts in the first second and the floor ripping shot holes, as well as the rim holes are visible. All the time stages are clearly recognizable with all three devices. In the case of time recording from the interferometric sensor, the effect of the near zone is evident already in the first second (cut), when, in most cases, the cut generally has the highest charge density (more explosive per time stage) and, thus, also the greatest amount of energy it exerts on the surrounding rock mass [48].

**Figure 14:**
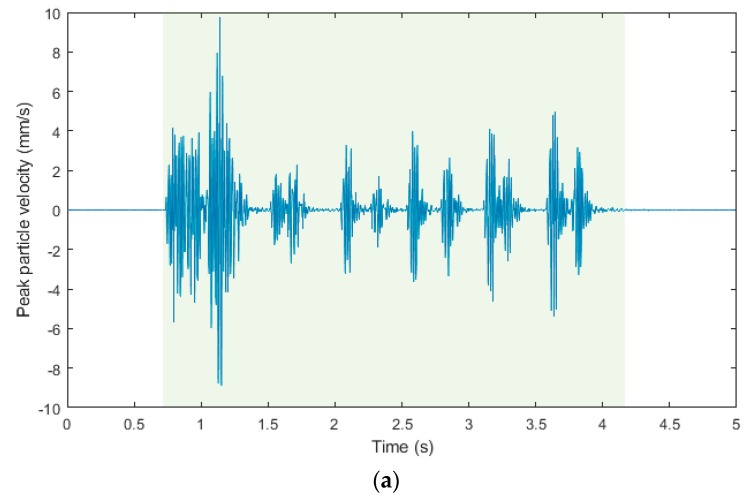

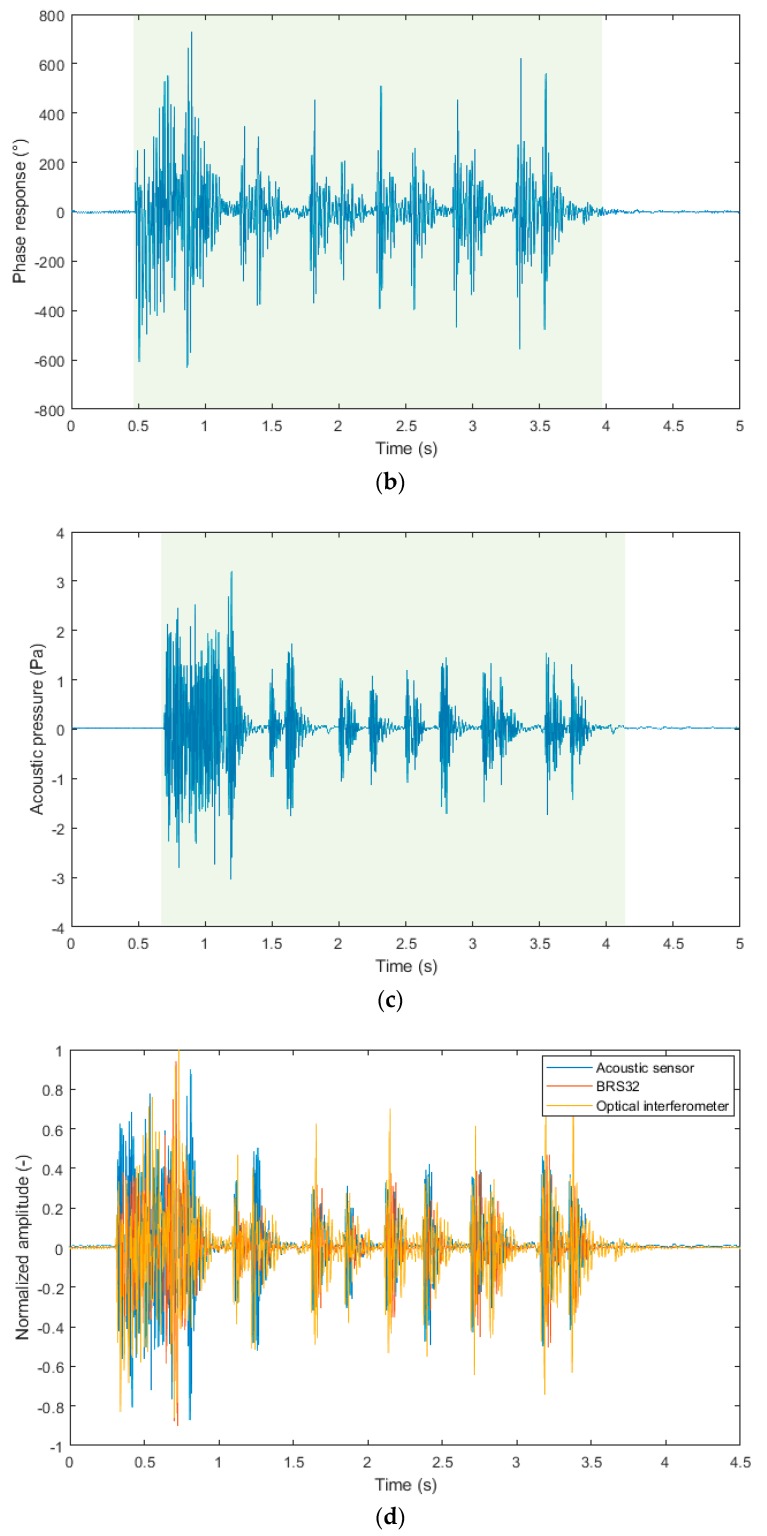
The waveform images from the blasting operation recording of 26 April 2019: (**a**) Seismic apparatus BRS32; (**b**) interferometric sensor; (**c**) acoustic sensor; (**d**) graphical comparison of the three records described above in one graph.

Figure 14a–c shows time domain recordings from the blasting operation of 26 April 2019, which was located 29.8 m from the measuring station. The blasting operation recorded was again of the same length - three and a half seconds, please see the light green background, which was detected by all the three devices. Figure 14d, again for better clarity, illustrates a graphical comparison of the three records described above in one graph. The timing was identical to the blasting operation of 8 April 2019. Hence, forty pieces of DeM detonators with explosives in the total amount of 9 kg and ten pieces of DeD detonators with explosives in the total amount of 3 kg were used for timing. Both the shot of the cuts in the first second and the floor ripping shot holes, as well as the rim holes are visible in the recording. All the time stages of the blasting operation are clearly recognizable in all three devices, and the near-zone manifestation is again identifiable only in the time recording obtained from the interferometric sensor in the first second.

**Figure 15:**
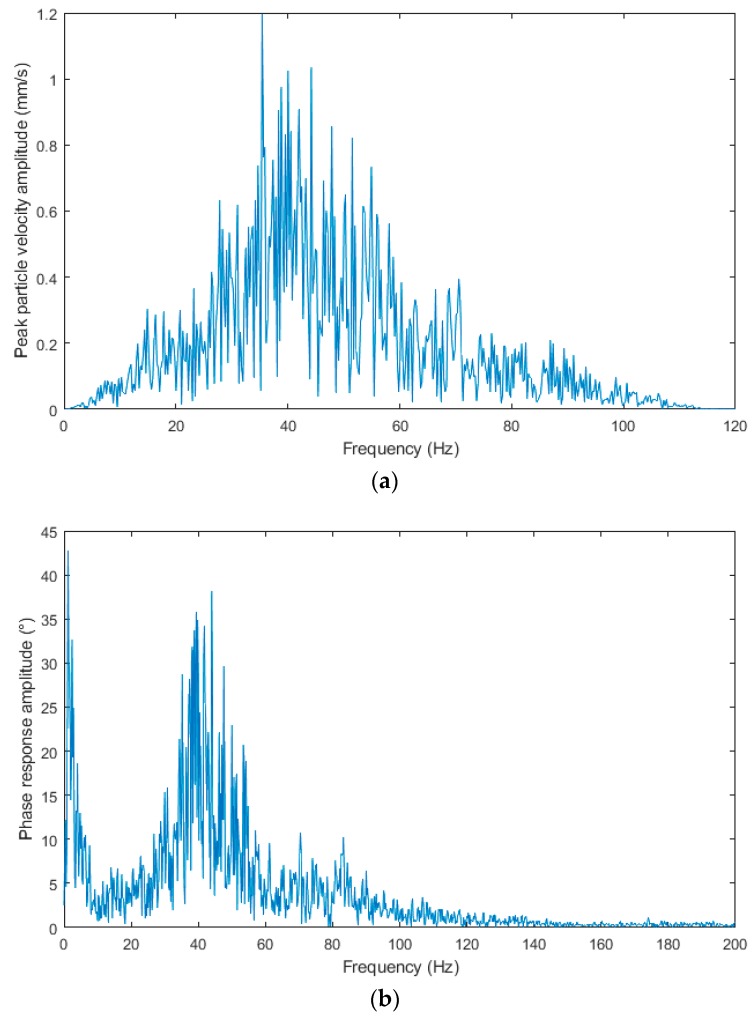

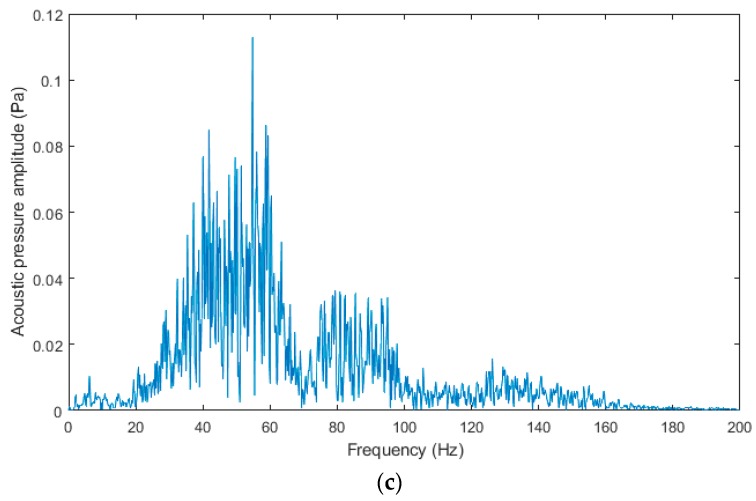
The frequency spectra from the blasting operation recording of 11 March 2019: (**a**) Seismic apparatus BRS32; (**b**) interferometric sensor; (**c**) acoustic sensor.

Figure 15a–c shows the frequency spectra from the blasting operation recording of 11 March 2019. The spectrum was always calculated per 5 s of the signal (the window length was, however, different for each sensor type because of a different sampling rate) and the rectangular window function was used. The predominant frequency in all the three spectra is in the range from 30 to 60 Hz with a more pronounced peak in the range from about 35 to 50 Hz. In the spectrum obtained from the interferometric sensor, a sharp and pronounced peak is also clearly visible in the region of very low frequencies, which corresponds to the measurements in the near zone, see above (elastic deformation of rock mass).

**Figure 16:**
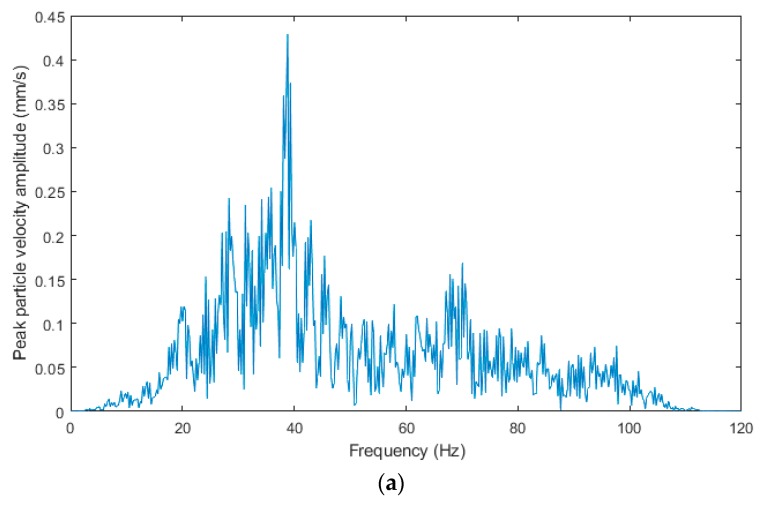

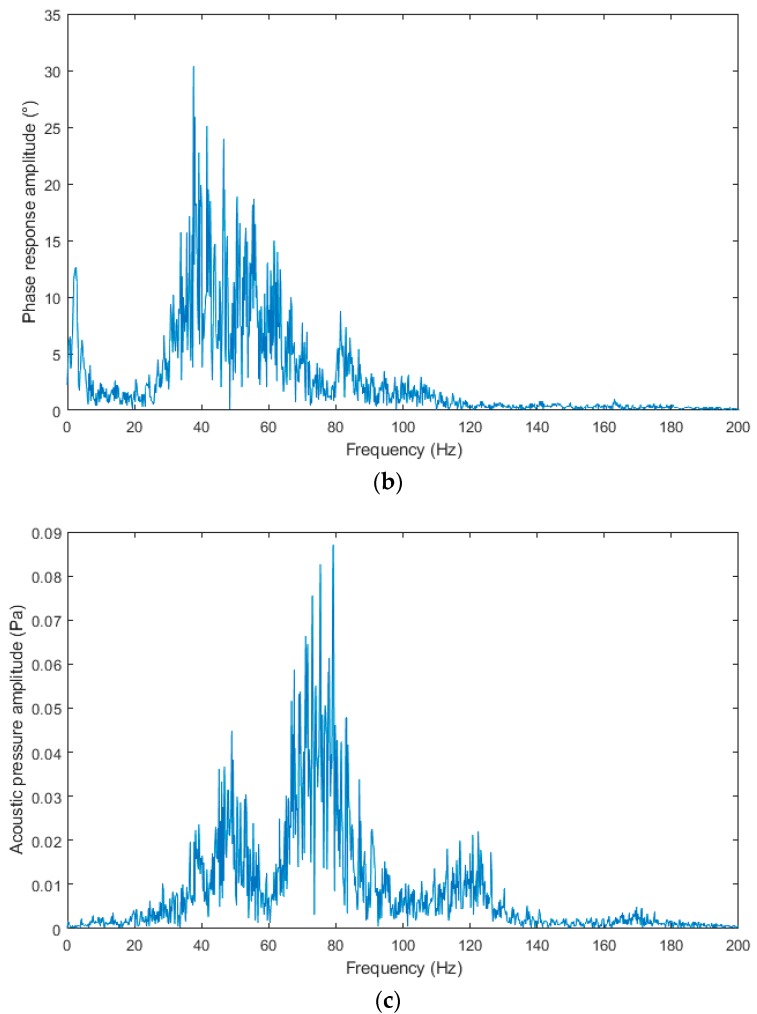
The frequency spectra from the blasting operation recording of 8 April 2019: (**a**) Seismic apparatus BRS32; (**b**) interferometric sensor; (**c**) acoustic sensor.

Figure 16a–c shows the frequency spectra from the blasting operation recording of 8 April 2019. The predominant frequency in the spectra is in the range from 30 to 80 Hz with more pronounced peaks in the range from about 50 to 80 Hz. In the spectrum obtained from the interferometric sensor, not such a pronounced peak is again visible in the region of low frequencies as a manifestation of near-zone measurements.

**Figure 17:**
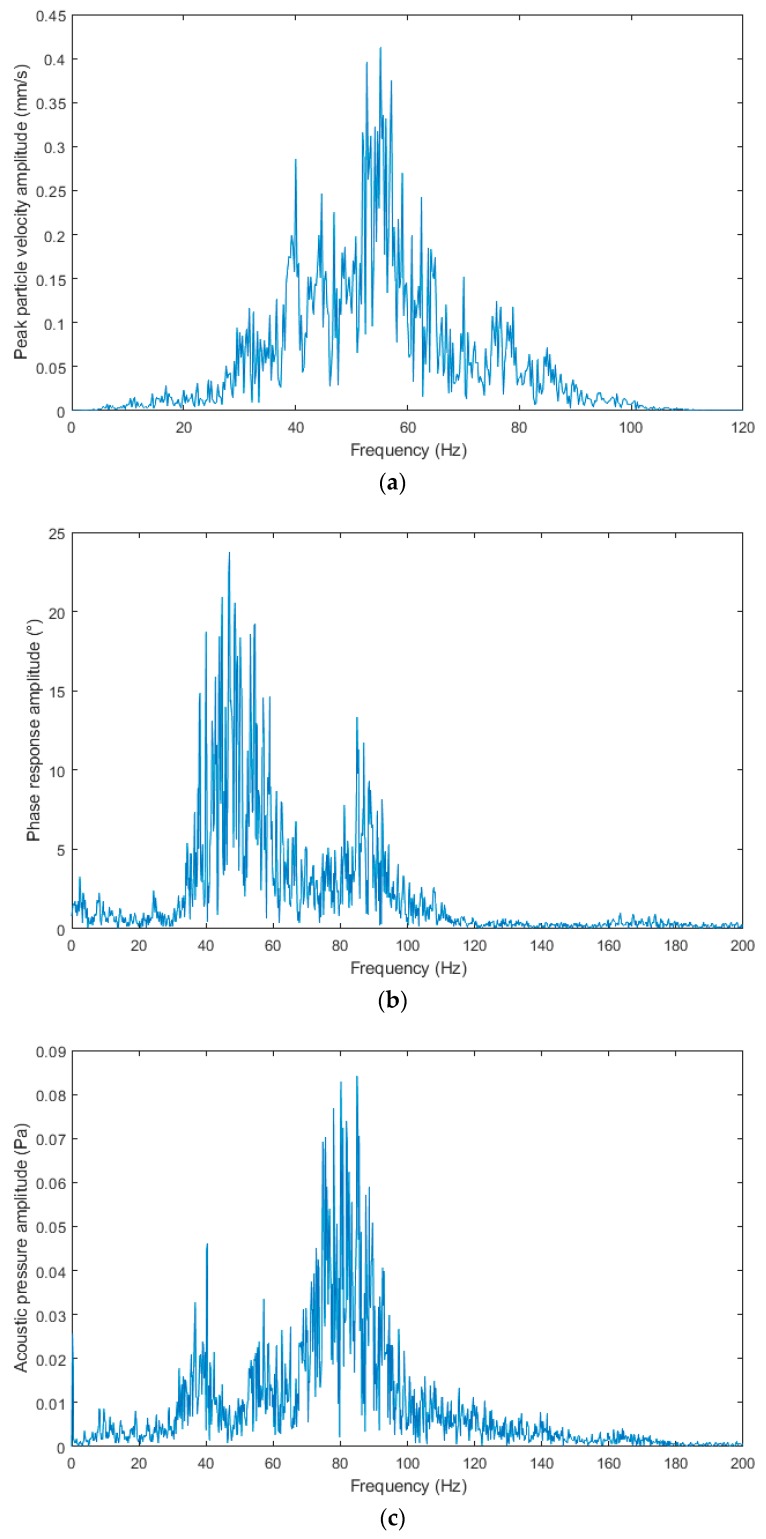
The frequency spectra from the blasting operation recording of 26 April 2019: (**a**) Seismic apparatus BRS32; (**b**) interferometric sensor; (**c**) acoustic sensor.

Figure 17a–c shows the frequency spectra from the blasting operation recording of 26 April 2019. The spectra are very similar to those of the previous blasting operation, which had the same blast geometry and the distance difference of 7 m. The predominant frequency in the spectra is in the range from 30 to 100 Hz with more pronounced peaks in the range of 40, 60 and 80 Hz. In the spectrum from the interferometric sensor, the peak in the low frequency region almost disappeared.

All the frequency spectra presented correspond to the frequency content of blasting operation manifestations and indicate that they can substitute standard seismic instrumentation in the frequency domain, please see Table 1. The more pronounced peaks shift, slightly, into the higher frequency range with increasing distance from the measuring station. The differences in the specific dominant peaks are caused by the design of the individual first prototypes of measuring equipment. The summarization of the frequencies obtained is shown in Table 1. The interferometric sensor is labeled as INTS, and the acoustic sensor is labeled as ACOS.

## 4. Discussion

The basic tasks of seismic and geotechnical engineering in the field of blasting operations are to assess the impact of tunnel excavation on the surrounding buildings and to determine the attenuation parameters of the surrounding rock environment. The assessment of the impact of the excavation on building structures is carried out in both the amplitude and frequency areas based on station measurements and the evaluation is conducted according to valid standards [49,50,51]. Determination of the attenuation of the environment is performed by profile measurements and is evaluated on the basis of the maximum amplitude of the oscillation and the reduced distance. In general, exponential dependence is accepted for damped oscillation, but power dependence is the most immediate in the near zone [52,53].

So far, the above mentioned can only be performed using standard seismic instrumentation. However, the pilot study presented shows that experimentally developed devices operating on other physical principles than a typical seismic station are capable of registering blasting operation manifestations in both the amplitude and frequency domains. In the amplitude domain, all the recordings of each blasting operation have the same length when compared to one another, and the length corresponds to the actual blasting operation, and a significant match of all the maxima corresponding to the individual time stages of the blasting operation can be observed.

In the frequency domain, there is a significant agreement in the width of the band measured, which corresponds to the blasting operation effects [7], and also in the maxima where the interferometric sensor has a greater match compared to the amplitude domain. With an acoustic sensor, the maxima are shifted to higher frequencies, which may be caused both by the design and the alignment of the sensor, wherein the device is primarily designed for installation in a rock mass.

A sensor based on the acoustic tube measures the displacement of a mass point (that’s mean the influence of surrounding material motion (mechanical waves) of the surrounding material). The propagation of the longitudinal wave in the geological subsoil affects the pressure inside the acoustic tube, due to changes in the volume of the tube. This change is recorded using the microphone. The disadvantage of this method lies in the measurement of scalar, as well as in the case of the used optical sensor. When measuring the longitudinal wave as a function of pressure in the tube, we lose its direction information.

As can be seen from the principle of sensor function, the system is susceptible to acoustic interference in comparison with the optical system. In the event of failure of the integrity of the acoustic tube, pressure leakage, and permeation of acoustic vibration into the conduit will occur.

It is possible to measure vibrations along the entire length of the sensor (acoustic tube). Assuming that the rate of excitement propagation in a low-density substance (normal atmosphere inside the tube) is negligible in comparison to excitement propagation in a high-density substance (the tube and the surrounding rock), the excitement, thus, occurs at the same time along the entire length of the tube and there is a uniform pressure change, due to the incoming excitement.

The main parameter of the acoustic sensor is the modulus of elasticity, which allows the sensor to function and converts longitudinal waves into pressure changes. These pressure changes propagate inside the acoustic tube and can be further influenced by its length and spatial distribution. A spiral-shaped tube placed in a 20 cm square box was used. This design was chosen to prevent the formation of secondary pressure waves and its possible interference. The elastic tube of the sensor can be elongated using a rigid tube, but this creates a time delay for the passage of the pressure wave through the rigid tube. 

Unlike the optical system, the acoustic system sensor can be deformed reversibly. The sensor is also resistant, to a certain extent, to mechanical damage of a non-destructive nature, which does not damage its airtight case.

The main reason for the wide frequency range are the parameters of the microphone. It is the most important sensor component. The tube behaves like a membrane, which reacts with its entire surface to the movements of the surrounding material. This movement modulates the volume of the tube and, consequently, the pressure inside it. As a result of the design, the pressure changes only in front of the membrane, in the flexible tube.

As mentioned above, in the case of the interferometric sensor, we do not measure in three components either, so we do not receive information about the spatial motion of the mass point. The presence of temperature drift was captured in some recordings; see the presence of very low frequencies in the measured spectrum in Figure 15b. This limits the lower cut-off frequency of the fiber-optic sensor; due to the sufficient sensitivity, it will be solved by slight shortening of the interferometer arms; the sensor sensitivity has proved to be very good.

In case of the interferometric sensor, it is necessary to employ a demodulation technique, and the passive demodulation used requires several optical fibers; it is necessary to use multi-fiber cables (which is not a problem, due to the dimensions of the optical fiber in the cable).

The advantages of the optical system include a wider frequency range—it is also possible to measure an acoustic manifestation of blasting operations. Furthermore, the passive operation of the interferometric sensor and the evaluation unit with optoelectronics can be located at a considerable distance (even several km) from the measurement site. This enables placement of the sensor, for example, in explosive environments or in highly electromagnetically disturbed environments. There is a possibility to share some system components when measuring with several sensors simultaneously or in several axes (e.g., laser diodes, AD converters, evaluation unit). A sensor network can, thus, be effectively created, which is particularly advantageous in the case of continuous monitoring. 

The advantage of experimentally developed sensors from the perspective of a broader frequency range is the possibility of measuring both vibrations and acoustic noise by a single device. Pilot research is currently underway, where building constructions are monitored by measuring vibration, and acoustic noise from large-scale blasting operations carried out in quarries using standard instrumentation intended for this purpose (a seismic station with the range 4.5 Hz to 100 Hz and acoustic meter with linear range 0.5 Hz to 20 kHz) and also fiber-optical interferometer. 

A sample price is also stated here, which is only an example of the final price of the seismic station and the unit prices on the basis of which the experimentally developed sensors were created. The price does not include work, sale, etc. 

Table 2 below compares the seismic station, the interferometric sensor and the acoustic sensor. Basic parameters, such as bandwidth, sampling frequency, dimensions, weight, etc. are described, including the sample price of the system (i.e., sensor + evaluation unit)). 

## 5. Conclusions

The paper presents a pilot study dealing with alternative approaches to measuring dynamic manifestations of blasting operations-vibration. Three types of measuring devices operating on three different physical principles were installed on the surface within three experimental measurements on a real construction site, where small-scale blasting operations were performed as part of cyclic excavations. The first device was an experimentally developed interferometric sensor, the second one was a unique acoustic sensor for vibration registration, and a standard seismic station BRS32 was used as an etalon. The measurement results were evaluated primarily in the frequency and also in the amplitude domain. In the frequency domain, both the spectrum character and the bandwidth and peaks were matched, and, in the amplitude domain, there was a match in the character and the length of the recording. This pilot study points out that, according to ISO 4866:2010 Mechanical Vibration and Shock [7], the results obtained correspond, by their frequency content, to the manifestations of blasting operations and indicate that, at least in the frequency domain, the experimentally developed devices can substitute standard seismic instrumentation. The following research will focus primarily on the time domain, but also on large-scale blasting operations, measurements outside the near zone, as well as measurements on structural elements of buildings. 

The presented article is aimed primarily at the scientific community, which deals with the development of new equipment in the field of vibration measurement with practical application in the field of seismic blasting and mechanical vibrations measurement in general.

## Figures and Tables

**Table 1:** Summarization of the frequencies obtained from the three measurements.

Date	Bandwidth (Hz)			Dominant Component (Hz)		
	BRS 32	INTS	ACOS	BRS 32	INTS	ACOS
11 March 2019	30–60	30–58	30–65	35	45	55
8 April 2019	39–75	30–70	30–80	48	41	75
26 April 2019	30–79	35–95	35–98	52	49	80

**Table 2:** Comparison of the basic parameters of the seismic station, the interferometric sensor and the acoustic sensor.

Type of Sensor	Bandwidth (Hz)	Sampling Frequency (Hz)	Size (mm)	Weight (kg)	Price (USD)
**BRS 32**	4.5–100	125–500	270 × 245 × 130	4.6	3100
**INTS**	2–5000	10,000	500 × 500 × 350	8	1700
**ACOS**	20–18,000	5000–50,000	80 × 80 × 30	0.3	1500
